# Electrocatalytic study of NiO-MOF with activated carbon composites for methanol oxidation reaction

**DOI:** 10.1038/s41598-021-96794-7

**Published:** 2021-08-25

**Authors:** Saadia Hanif, Naseem Iqbal, Tayyaba Noor, Neelam Zaman, K. Vignarooban

**Affiliations:** 1 USPCAS-E, National University of Sciences and Technology, Islamabad, 44000 Pakistan; 2 SCME, National University of Sciences and Technology, Islamabad, 44000 Pakistan; 3 Department of Physics, Faculty of Science, University of Jaffna, Jaffna, 40000 Sri Lanka

**Keywords:** Electrocatalysis, Fuel cells

## Abstract

In this work, the methanol oxidation reaction is investigated on Ni based metal organic frameworks (MOF) and its composites with biomass derived activated carbon. NiO-MOF and composites with activated carbon were synthesized using hydrothermal method. SEM, EDX, and XRD, FTIR, TGA techniques were used for characterization of composites. The electrochemical activity of catalysts for oxidation of methanol was tested using cyclic voltammetry (CV) in 1 M KOH and 3 M CH_3_OH on glassy carbon electrode in three electrode setup. The electrochemical performance shows the effect of activated carbon concentration on methanol oxidation. The electro-oxidation catalyzed by NiO-MOF with activated carbon (40 mg) composite exhibits a peak current density of 182.72 mA/cm^2^ at 0.89 V potential with a scan rate of 50 mV/s making it a potential catalyst for electrocatalysis of methanol.

## Introduction

The global energy crisis, impending energy reserves and the deteriorating environment calls for alternative green energy. In addition, sustainable and proficient energy conversion technologies are extremely needed because of the rapid diminution of fossil fuels and the immense apprehension of human society about environmental pollution. In order for intermittent energy generation technologies to strengthen their foothold, energy storage solutions need to become better performing and economically viable. Implementation of fuel cell technologies has been greatly encouraged for suppressing the emission of carbon dioxide^1^. Various sources have been explored to deliver sustainable, environmentally friendly and inexpensive energy. Fuel cells deem ideal candidates meeting all these requirements^2^.

Various materials can be used as catalysts such as platinum and platinum alloys^3,4^. However, platinum and its alloys being expensive metals increase the overall cost of the fuel cell. In the recent years research has been shifted finding alternative catalysts with high activity, high stability and low cost^5^. Zhao et al. reported star like Pt-Cu/rGO nanoparticles as electrocatalysts for methanol oxidation. The reported nanoparticles show a density of 40.2 mA/mg at 50 mV/s scan rate in 1 M CH_3_OH and 0.5 M of H_2_SO_4_^6^. Moreover, Qiu et al. reported platinum and graphene nanocomposites for methanol oxidation. The reported composite show 2.53 mA/cm^2^ current density at a scan rate of 10 mV/s in 1 M H_2_SO_4_ and 2 M CH_3_OH solution^7^. Recently, a new family of porous solids called porous coordination polymers or metal organic frameworks (MOFs) are becoming popular as electrocatalysts for fuel cells^8^. Typically, MOFs are formed by combining metals and ligands through coordination bond. They are characterized by maximum degree of crystallinity, porosity; high surface area and pore size clearly going beyond that of other porous materials^9^. Researchers have discovered that small alteration to functional groups located on the linkers can greatly enhance potential properties of MOFs; for example in drug delivery, heterogeneous catalysis, gas storage, separation and so on^10^. Application of MOFs in catalysis are determined on the basis of successfully developed synthetic principles; which include catalyst encapsulation in framework, stabilization of catalytically functioning nanosized particles, heterogenization of corresponding catalysis, post synthesis insertion of catalytic metal site and alliance of catalysis with chemical separation^11^.

Carbon based electrocatalysts have shown promising portfolios over the years. They provide good mechanical strength, increased active surface area, and enhanced conductivity at a lower cost. The problem however is their low activity. So, composites of carbon based materials are made with other established electrochemically active materials such as MOFs. MOFs are crystalline materials with high porosity and high activity^12,13^. Their activity and stability can be enhanced by the incorporation of carbon^14^. Mehek et al. reported Co-MOF-71, and Co-MOF-71/GO composites. Their appreciable high current density makes them promising substitutes to the other expensive electrocatalysts for various fuel cell applications. They show current density of 29.1 mA/cm^2^ at 50 mV/s. The appreciable electrocatalytic activity and stability of prepared material for methanol oxidation was associated with the corresponding effect of Co-MOF and graphene oxide^15^. Noor et al. reported NiO-MOF and 1–5, 8 wt% rGO NiO-MOF composites. Among all the composites studied by them, 5wt% rGO NiO-MOF show superlative current density of 285.73 mA/cm^2^ at 50 mV/s in 3 M methanol and 1 M NaOH solution^16^. Yaqoob et al. reported Ni-BTC MOF and its composites of 1–5 and 8 wt% rGO for methanol oxidation. The best results were obtained for Ni-BTC/4 wt% rGO of about 200.22 mA/cm^2^ given 0.69 V and 50 mV/s in 2 M CH_3_OH solution with 1 M NaOH as support electrolyte. Besides, with the increase in the amount of catalyst, the current density is gradually increased until 2 mg, which was recognized as an ideal concentration for electrochemical testing^17^.

In this work, we report nickel based metal organic framework and its composite with biomass derived activated carbon (AC) as efficient catalytic material for methanol oxidation reaction. The work focuses on electrocatalytic studies of NiO-MOF and its composites with AC in alkaline media. All the prepared series of metal organic framework composites exhibit exceptional high electrocatalytic activity with AC to enhance the conductivity of catalyst.

## Experimental

All reagents and chemicals were purchased from Sigma-Aldrich with superlative quality and used without further alteration. The chemicals used were nickel nitrate hexa hydrate (NiNO_3_.6H_2_O), benzene di-carboxylic acid (terephthalic acid), *N*,*N*-dimethylformamide (DMF), triethylamine (TEA) and phosphoric acid (H_3_PO_4_).

### Synthesis of NiO-MOF

NiO-MOF was synthesized from the already reported method^17^. In 100 ml of DMF and 0.41 g of terephthalic acid was dissolved by stirring. Then, few drops of triethyl amine was added in the solution. The Nickel nitrate hexahydrate (0.61 g) was then added, and the resulting solution was stirred for 30 min. The prepared solution was poured into an autoclave (Teflon lined) and heated at 120 °C for 24 h in an oven. The crystals of NiO-MOF were collected through filtration, repeatedly washed with DMF to washout extra organic materials followed by drying in vacuum oven at 80 °C.

### Synthesis of activated carbon from waste

Fallen waste leaves of Lantana plant were collected from garden and washed thoroughly to remove all impurities. After proper drying, leaves were grinded to reduce its size until it can pass through a mesh size of 200 microns. Then, the obtained powder sample was dissolved in a solution of diluted phosphoric acid (H_3_PO_4_) for 16 h. It was then calcined at a temperature of 550 °C to activate it, as this temperature results in increasing carbon burn-off and evaluation of volatiles from the samples^18^. Acid wash/neutralization was done with distilled water to remove any unreacted acid and neutralize the pH of the solution up to 6–7. Resulting AC was then dried and stored for further use^15^. The plant material used in this research comply with relevant institutional, national, and international guidelines and legislation.

### Synthesis of NiO-MOF @ AC composites

**Figure 1:**
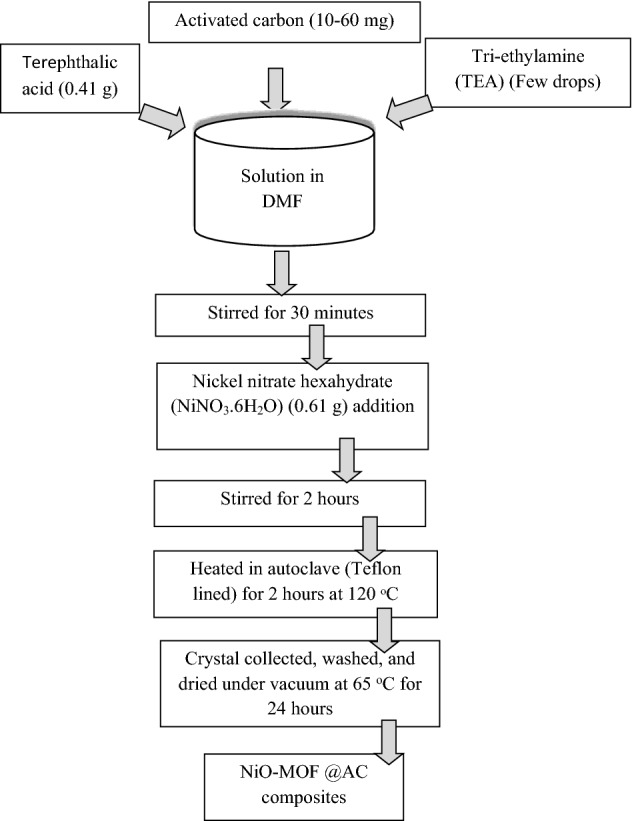
Schematic of preparation steps for NiO-MOF @AC composites.

In 100 ml of DMF, terephthalic acid (0.41 g) was dissolved by stirring. Then, few drops of triethyl amine was added in the solution. The Nickel nitrate hexahydrate (0.61 g) was then added in the solution and the resulting solution was stirred for 30 min. Activated carbon (10–60 mg) was added into the solution and stirred for 2 h. The prepared solution was poured into an autoclave (Teflon lined) and heated at 120 °C for 24 h in an oven. The crystals of NiO-MOF@AC were collected through filtration, repeatedly washed with DMF to washout extra organic materials followed by drying in vacuum oven at 80 °C Fig. 1 shows the schematic of preparation steps for NiO-MOF @AC composites.

### Characterization of catalysts and elemental analysis

Scanning Electron Microscopy (SEM) was done with VEGA3 TESCON at different resolutions and voltage of 20 kV to study surface morphologies. Energy dispersive spectroscopy equipped with SEM was also used to study the elemental analysis of composite at three different spots. X-ray powder diffractometer (XRD STOE Germany) was used to observe the crystal lattice of composite. XRD machine is linked with a computer interface having Cu Kα at λ = 1.5418 Å and the diffraction angle 2θ was varied at a range of 5–70° with a step size of 4° s^−1^. With the help of Perkin spectrum 100 FT-IR spectrophotometer Metal ligand co-ordination was proved at the wave number range of 500–4000 cm^−1^. Thermo-gravimetric analysis (TGA) was employed by means of DTG-60H to test the thermal stability of composites such as NiO-MOFAC (40 mg) in a nitrogen atmosphere.

### Electrochemical evaluation

The electrochemical measurements were done by using Gamry instrument Reference 3000/3000 equipped with data acquisition software version of 7.06. Electrochemical activity of prepared catalysts was tested via cyclic voltammetry (CV) in 1 M KOH and 3 M CH_3_OH by using three electrode set-up. In this set-up, glassy carbon, silver/silver chloride (Ag/AgCl) and Pt wire were used as the working electrode, reference electrode, and the counter electrode, respectively. Methanol and KOH were used as a fuel and supporting electrolyte, respectively. 5wt% Nafion solution and ethanol were used as the binding agent and solvent, respectively. The CV study was first performed with glassy carbon electrode (GCE) without any catalytic material and next the set-up was run with GCE coated with catalytic material. Electrochemical impedance spectroscopic (EIS) study was conducted by using same three electrode set-up mentioned above in 3 M CH_3_OH and 1 M KOH, under potentiostatic mode. The range of the frequency was set from 10 to 40 kHz with an amplitude of 0.015 V.

### Electrode fabrication

For CV and EIS measurements, a catalyst ink was prepared by mixing the catalytic material (2 mg), ethanol (100 μL) and Nafion (5 wt% solution) (20 μL). The mixture was mixed thoroughly, and then as prepared catalyst ink (15 µL) was deposited on GCE by micropipette. Before each electrochemical measurement, the modified electrode was dried at room temperature.

## Results and discussion

**Figure 2:**
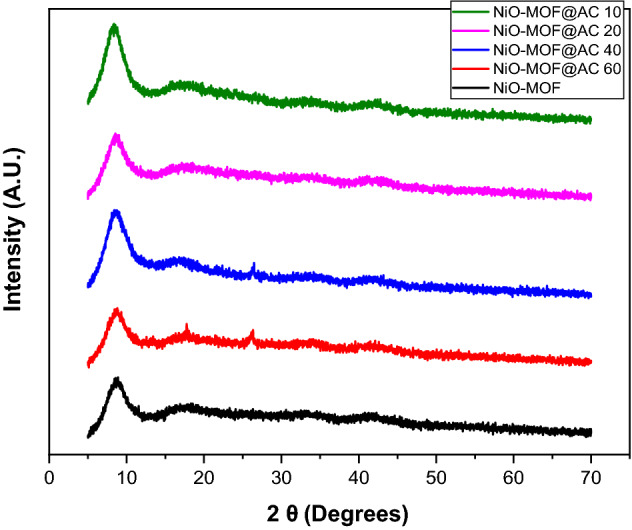
Powder XRD of NiO-MOF and its composites with activated carbon.

For detailed crystal structure, XRD study was carried out for all NiO-MOF and activated carbon composites, i.e., NiO-MOF@AC 10, NiO-MOF@AC 20, NiO-MOF@AC 40, NiO-MOF@AC 60 (Fig. 2). All the composites have retained the characteristic peak of NiO-MOF at 10 degree. A low intensity peak at 26 degrees corresponds to the carbon and a characteristic peak at 44.7 corresponds to nickel oxide (NiO JCPDS card No. #04-0835)^19–21^.

**Figure 3:**
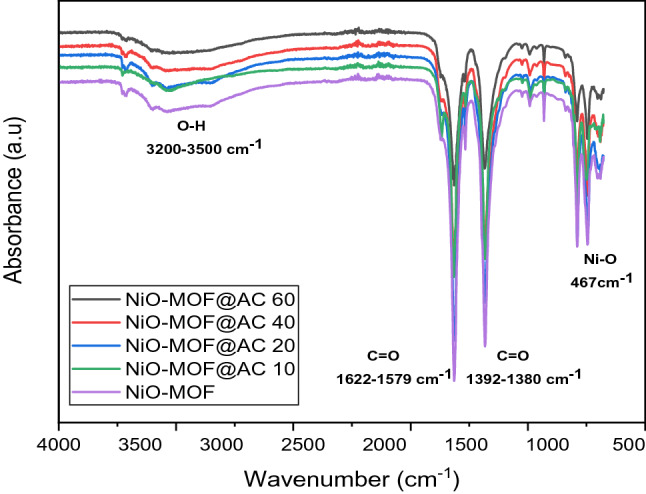
FTIR spectra of NiO-MOF and its composites with activated carbon.

Figure 3 shows the FTIR absorbance spectra of NiO-MOF, and its activated carbon based composites such as NiO-MOF@AC 10, NiO-MOF@AC 20, NiO-MOF@AC 40 and NiO-MOF@AC 60. In all the prepared electrocatalysts, the presence of broad peak between 3200 and 3500cm^1^ shows the hydroxyl group (OH^−1^) presence and the sharp band at 1392–1380 cm^−1^ and 1622–1579 cm^−1^, shows the presence of C=O symmetric and asymmetric stretching vibrations. Further, the deprotonation of functional group, i.e. –COOH in benzene dicarboxylic acid is confirmed via absence of strong absorption peak at 1715–1680 cm^−1^. Besides, Ni–O stretching band appear at 467 cm^−1^^30^, proves the metal–ligand linkage. Thus, the absorption spectra of the prepared composites agree with the successful synthesis of NiO-MOF and its activated carbon based composites^31^.

**Figure 4:**
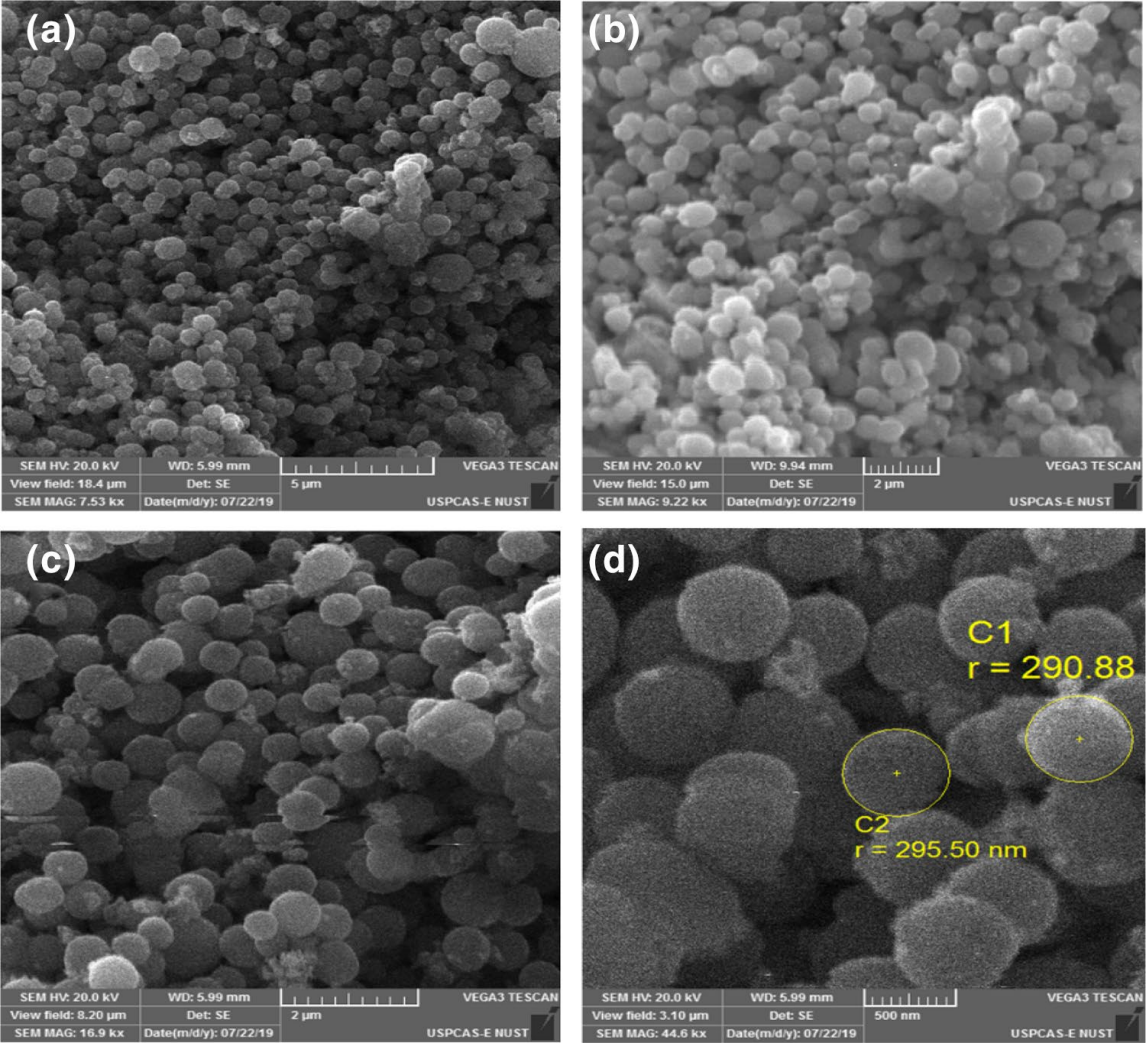
SEM images of NiO-MOF@AC 40.

Morphology of NiO-MOF@AC 40 was studied with the help of SEM and shown in Fig. 4. The SEM images show spherical structure with a mean diameter of 600 nm for the NiO-MOF@AC 40. The activated carbon particles can also be seen incorporated between the spherical structures of NiO-MOF. Table 1 shows the EDX analysis of NiO-MOF and its composites with activated carbon such as NiO-MOF@AC 10, NiO-MOF@AC 20, NiO-MOF@AC 40 and NiO-MOF@AC 60 shows the presence of oxygen and carbon along with nickel. Likewise, the elemental content of carbon increases consequently with activated carbon, assigning the efficacious assimilation of activated carbon in MOF.

**Table 1:** EDX analysis of NiO-MOF and its composites with activated carbon.

Sample/elements	NiO-MOF	NiO-MOF@AC 10	NiO-MOF@AC 20	NiO-MOF@AC 40	NiO-MOF@AC 60
C wt%	33.01	41.78	44.87	46.19	49.88
O wt%	41.02	40.20	39.12	38.83	37.93
Ni wt%	25.97	18.02	16.01	14.98	12.19

**Figure 5:**
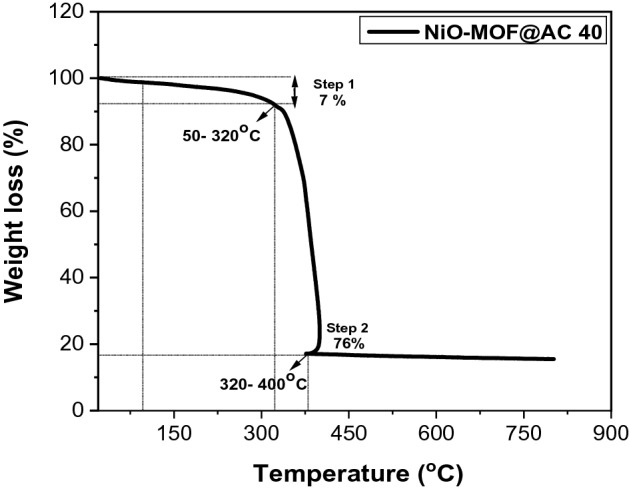
TGA analysis of NiO-MOF@AC 40 measured under N_2_ environment.

Figure 5 shows the TGA data of prepared composite NiO-MOF@AC 40 measured in a nitrogen atmosphere. TGA curve illustrate the weight loss of 7% till 320 °C and can be attributed to the evaporation of adsorbed water molecules roughly between 50 and 320 °C. In the temperature range of 320–400 °C, the second weight loss is appearing that is up to 76% ensuing from the decomposition of organic ligands. After 400 °C, the weight of the NiO-MOF@AC 40 is found to be relatively stable. The elevated thermal stability of prepared activated carbon based composite can be ascribed to the coordinative linked interpenetrated framework^22,23^.

**Figure 6:**
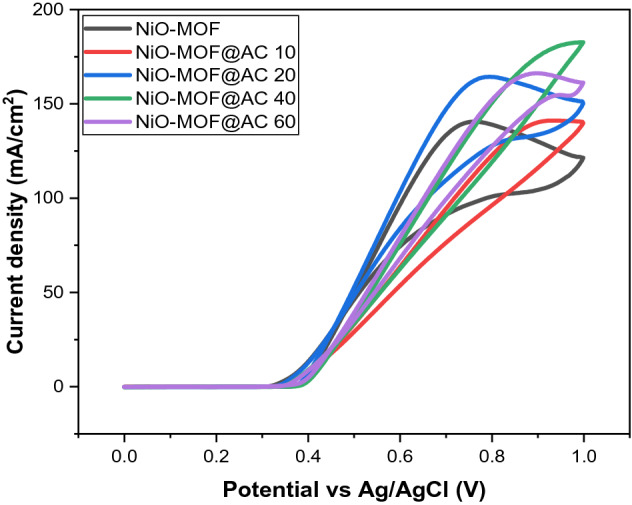
Cyclic voltammogram of NiO-MOF and composites with activated carbon in 1 M KOH and 3 M methanol.

Electrochemical performance of NiO-MOF@AC composites was tested by using cyclic voltammetry in 1 M KOH and 3 M CH_3_OH at 50 mV/s for oxidation of methanol. The NiO-MOF with activated carbon composites exhibited more catalytic activity towards methanol oxidation reaction (see Fig. 6). In case of NiO-MOF modified GCE, the peak current density was 140 mA/cm^2^ and by the addition of activated carbon, the peak current density increases gradually. The sample with greater concentration of activated carbon (40 mg) shows highest peak current density of 182.71 mA/cm^2^ at 50 mV/s scan rate. The other composites show peak current density values of 142.12 and 164 mA/cm^2^ for 10 mg and 20 mg activated carbon, respectively at a scan rate of 50 mV/s. With further increase in the amount of activated carbon (60 mg), the peak current density decreases to 166.28 mA/cm^2^. In summary, it is suggested that current density can be enhanced by increased concentration of activated carbon to a certain limit; however, augmented concentration of activated carbon might have an undesirable consequence on catalytic activity too^24,25^*.* Moreover, the CV curves have different shape and peak position for NiO-MOF@AC composites, is because of activated carbon incomplete dispersion at the time of synthesis and also due to its high quantity usage the clustering of the catalyst occurs that results in covering of MOF surface^25^, thus impeding the active catalytic sites to carry out the reaction at low potential value and consequently charge transfer happen at high potential^15^.

**Figure 7:**
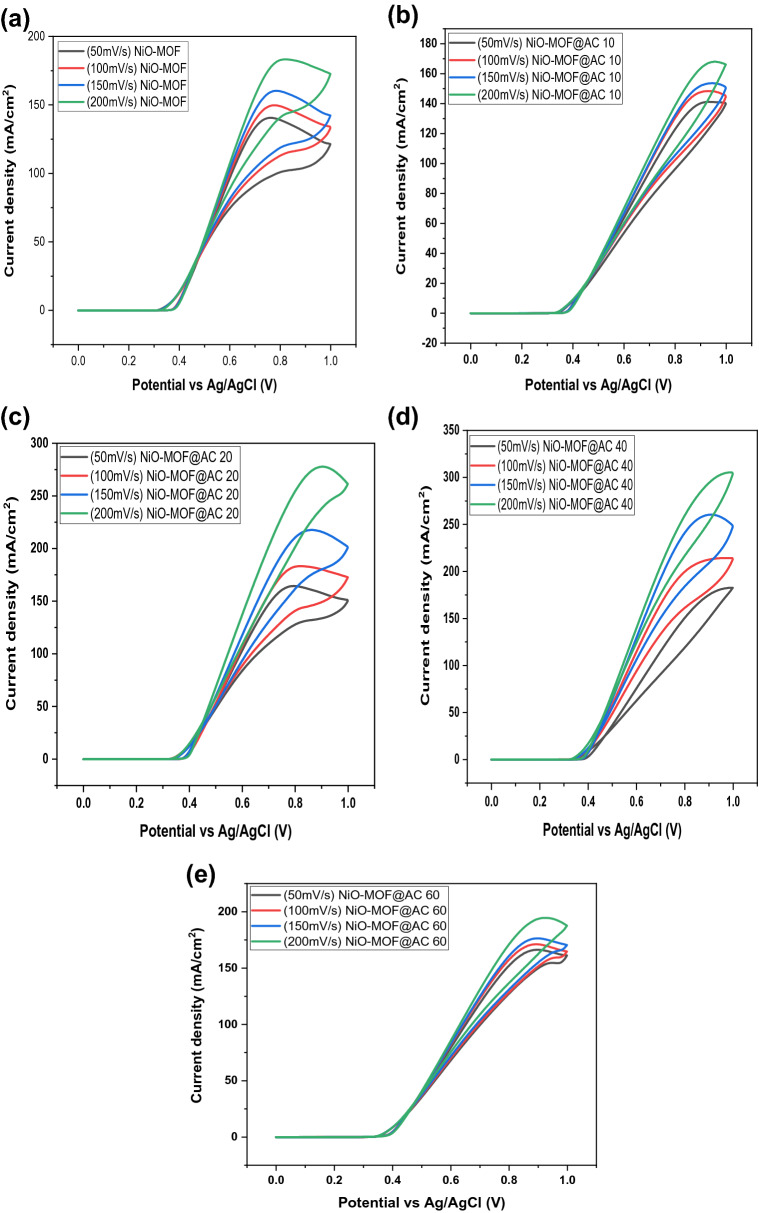
Cyclic voltammogram of (**a**) NiO-MOF, (**b**) NiO-MOF@AC 10, (**c**) NiO-MOF@AC 20, (**d**) NiO-MOF@AC 40, (**e**) NiO-MOF@AC 60.

The linear relationship of peak current densities of composites such as NiO-MOF@AC 10, NiO-MOF@AC 20, NiO-MOF@AC 40, NiO-MOF@AC 60 with scan rate is shown in Fig. 7. The figure describes the effect of scan rate, i.e., 50–200 mV/s on peak current density; with increasing scan rate, the peak current density also increases gradually. The reason for high current density at higher scan rate is due to non-electroactive species are not oxidized or reduced into the product^26^*.* Thus, high current density is obtained due to the formation of electroactive products. By analyzing the results, it can be figured out that increasing the scan rate i.e., 50 m–200 mV/s for all the composites can lead to corresponding increase in current density as well. This response of the catalytic material is associated with the enhanced extent of the reaction^27^. Table 2 compares the properties of prepared catalysts with other catalysts reported in the literature.

**Table 2:** Comparison of current densities, methanol concentrations and scan rates, with other electrocatalysts reported in the literature for methanol oxidations.

Sr. no	Catalyst	MeOH conc. (M)	Scan rate (mV/s)	Current density (mA/cm^2^)	Ref.
1	Co-MOF	3	50	10.4	^15^
2	5wt%GO/Co-MOF	3	50	29.10	^15^
3	ZnO_(40%)_/CeO_2(60%)_dots@CNFs	3	50	16.3	^28^
4	Cu-MOF	3	50	48.55	^29^
5	5wt% GO /Cu-MOF	3	50	120	^29^
10	NiO-MOF	3	50	140.59	This work
11	NiO-MOF@AC 10	3	50	142.12	This work
12	NiO-MOF@AC 20	3	50	164.42	This work
13	NiO-MOF@AC 40	3	50	182.71	This Work
14	NiO-MOF@AC 60	3	50	166.28	This work

**Figure 8:**
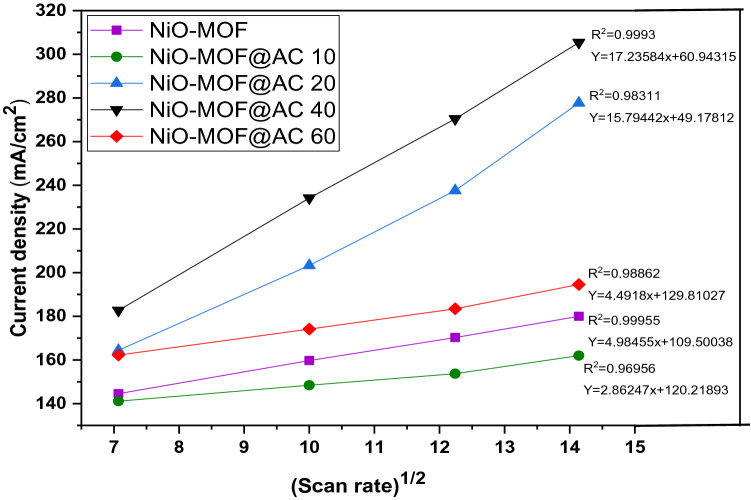
Plots of current density vs. square root of scan rate for NiO-MOF and its composites with activated carbon in 1 M KOH and 3 M methanol.

Peak current density and square root of scan rate is proportional to each other suggesting a diffusion-controlled process in correspondence with Randles–Sevick equation (given below with usual notations) as demonstrated in Fig. 8^30^.

$$ {\text{I}}_{{\text{p}}} = (2.99 \times 10^{5} ){\text{n}}\,(\upalpha {\text{n}}_{{\text{a}}} ){\text{ACD}}^{1/2} {\text{v}}^{1/2} $$

where Ip = Current in Amperes, n_α_ = No of electrons involved in rate determining step, A = Surface area of electrode (0.07065 cm^2^, n = No of electrons transferred in process, D = Diffusion co-efficient (cm^2^/s), C = Concentration in mole/cm^3^V = Scan rate in V/s.

Formula used to calculate α is as under

$$ {\text{Ep}} - {\text{Ep}}/2 = 1.857{\text{RT}}/{\text{nF}}\,\upalpha  $$

Ep = Peak potential, R = General gas constant (8.3143 J/k/mole), T = 298 k, n = No of electrons transferred in process, F = Farad’s constant (96,580), α = Charge transfer co-efficient.

For all the prepared catalysts, the diffusion coefficient (D) is calculated as 1.171 × 10^−3^, 1.174 × 10^−3^, 1.37 × 10^−3^, 1.78 × 10^−3^ and 1.42 × 10^−3^ for NiO- MOF, NiO-MOF@AC 10, NiO-MOF@AC 20, NiO-MOF@AC 40 and, NiO-MOF@AC 60, respectively. All these diffusion coefficient values keep the methanol oxidation that followed a diffusion controlled mechanism^31^.

**Figure 9:**
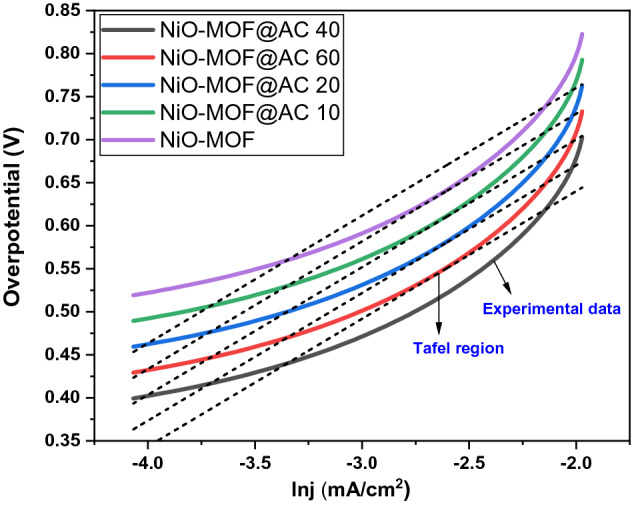
Tafel plots of NiO-MOF and its composites NiO-MOF@AC 10, NiO-MOF@AC 20, NiO-MOF@AC 40 and NiO-MOF@AC 60.

Tafel plots are represented in Fig. 9 derived from cyclic voltammogram. Overpotential is calculated via formula E–E_o_^32^. Commercial Pt/C catalyst shows these curves at or near 0.0 V but other materials give higher voltages. In this study, NiO-MOF@AC 40 composite displays a curve at lower overpotential. The improved overpotential at lower value represents its superior electrocatalytic activity, so less activation energy is required. With the composite formation with activated carbon, more surface improvement is achieved in the terms of pores. Due to these pores more ions can be incorporated at lower potentials. This results in increased kinetics with decreased activation energy for methanol oxidation^33,34^*.*

In addition, Tafel slopes at 0.55 V of potential were calculated for the study reaction kinetics of the catalytic process and listed in Table 3. Tafel slopes for NiO-MOF and its activated carbon based composites such as NiO-MOF@AC 10, NiO-MOF@AC 20, NiO-MOF@AC 40 and NiO-MOF@AC 60 are in the range of 121–185 mV/dec. The slopes at lower potential might suggest the first C–H bond breaking in methanol and first electron transfer that designates the rate-determining step^35,36^.

**Table 3:** Tafel slopes of NiO-MOF and its composites such as NiO-MOF@AC 10, NiO-MOF@AC 20, NiO-MOF@AC 40 and NiO-MOF@AC 60 at 0.55 V.

Catalyst	Tafel slopes at 0.55 V (mV/dec)
NiO-MOF	139
NiO-MOF@AC 10	156
NiO-MOF@AC 20	171
NiO-MOF@AC 40	121
NiO-MOF@AC 60	185

**Figure 10:**
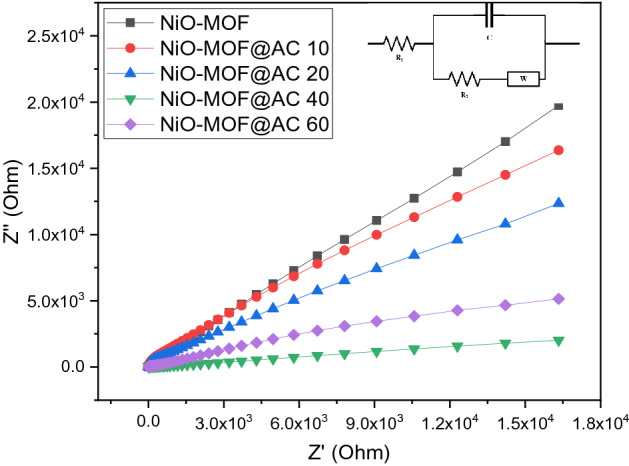
Nyquist plots of NiO-MOF and the composites with activated carbon in 1 M KOH and 3 M methanol recorded in potentiostatic mode with 50 mVs^−1^ scan rate, 40–100 kHz frequency range and 0.015 V of amplitude.

The electrochemical impedance spectroscopy (EIS) is another excellent technique to analyze the activity of modified electrode. Here, the electrochemical impedance was measured by using potentiostatic mode in the same three electrode set-up in 1 M KOH and 3 M methanol with bare and modified GCE and the corresponding Nyquist plots are shown in Fig. 10. These Nyquist plots show that as the amount of activated carbon in NiO-MOF increase, the charge transfer resistance decreases dramatically which is a clear indication of facilitation of charge transfer for the catalytic conversion of methanol^37–40^. The same has been concluded from voltammetry studies as mentioned above. The impedance results complement the voltametric findings. Moreover.

In order to extract EIS data, the model of electrochemical electrical circuit (EEC) with charge transfer resistance, solution resistance, capacitance, and Warburg diffusion co-efficient was best fitted on actual data extracted EIS data is given in Table 4. Firstly, in high to average frequency region a semicircle appear that not only provide the information about methanol partial oxidation but also give knowledge about Rct (a resistance at electrolyte/electrode boundary) and approve that system response is primarily controlled via charge transfer process by means of pores of electrode material. Moreover, the Warburg diffusion co-efficient is obtainable through slope (45°) in low frequency domain is associated with charge transfer activity and its minimal value prove that reaction go on all through diffusion controlled phenomena^38–40^.

**Table 4:** Electrochemical parameters from EIS data of NiO-MOF and its composites with activated carbon (NiO-MOF@AC 10, 20, 40, 60) in 1 M KOH and 3 M methanol solution.

Sr. no	Sample	Rs (Ohm)	R_ct_ (Ohm)	Warburg diffusion co-efficient (Ohm s^−1^)
1	NiO-MOF	53.54	184.3	9.4
2	NiO-MOF@AC 10	53.1	172.1	0.08
3	NiO-MOF@AC 20	52.9	168.1	0.05
4	NiO-MOF@AC 40	51.98	118.2	0.03
5	NiO-MOF@AC 60	53.2	123.2	0.04

**Figure 11:**
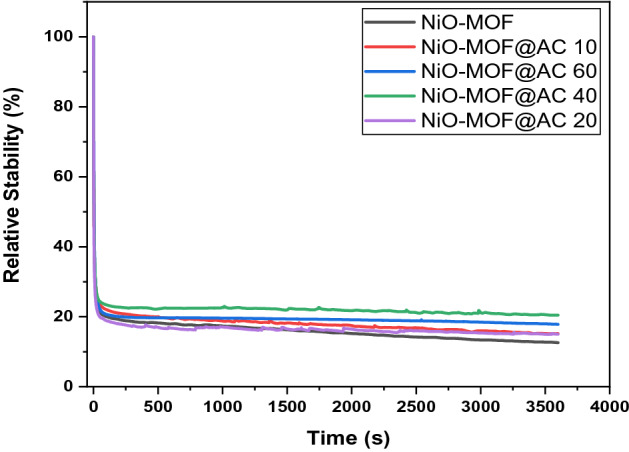
Relative stability curves of NiO-MOF and its composites with activated carbon in 1 M KOH and 3 M methanol.

Chronoamperometry was used to find the percentage stability of the prepared composites at 0.8 V of potential with similar electrode set-up in 1 M KOH and 3 M methanol solution and the results are shown in Fig. 11*.* It can be realized that quickly after the reaction starts, the current value falls to a specific value which is due to formation of an intermediate specie such as CO^41,42^ Afterward with the passage of time there is gradual decrease in current and up to 3600 s it attain a quasi-stationary state. This possibly because of methanol coverage on catalytic site at the start of the reaction but with the reaction progress an equilibrium layer of methanol develops on the surface of catalytic sites that slows down the overall process and carbon monoxide formation effects the stability of the all the prepared electrocatalysts. Among all the prepared composites, NiO-MOF@AC 40 in accordance with its high current density shows superior percentage stability (26%) in the set time period of 3600 s. Next to this, 23% for NiO-MOF@AC 60, 22% for NiO-MOF@AC 20, 21% for NiO-MOF@AC 10 and 20% for NiO-MOF. Efficient performances of all the composites clearly demonstrate that the incorporation of activated carbon in pure MOF to a certain extent will improve methanol oxidation process^41,42^.

## Conclusions

The NiO-MOF and its biomass-derived activated carbon composites were produced by less costly and environmentally favorable hydrothermal method. The synthesized materials were tested for electrocatalytic methanol oxidation reaction. The NiO-MOF composite (Ni-MOF@AC 40) exhibited best performance with highest current density of 182.71 mA/cm^2^ with lower over-potential values and higher stability. The NiO-MOF composite with activated carbon having non-noble metal makes it an inexpensive, but a potential candidate in the field of electrocatalysts for methanol oxidation to compete with Pt catalyst. The high current density and low cost makes it a promising alternative to the existing electrocatalyst in various fuel cell applications.
